# *In silico* screening for potential inhibitors from the phytocompounds of *Carica papaya* against Zika virus NS5 protein

**DOI:** 10.12688/f1000research.134956.2

**Published:** 2024-06-25

**Authors:** Kishore Krishna Kumaree, Naga Venkata Anusha Anthikapalli, Anchalee Prasansuklab

**Affiliations:** 1 College of Public Health Sciences, Chulalongkorn University, Bangkok, 10330, Thailand; 2 Natural Products for Neuroprotection and Anti-Ageing Research Unit, Chulalongkorn University, Bangkok, 10330, Thailand; 3 Department of Chemistry, Akkineni Nageswara Rao College, Gudivada, Andhra Pradesh, 521301, India

**Keywords:** Molecular docking, Zika virus, Papaya, AutoDoc Vina

## Abstract

**Background:**

The Zika virus (ZIKV) infection has emerged as a global health threat. The causal reasoning is that Zika infection is linked to the development of microcephaly in newborns and Guillain-Barré syndrome in adults. With no clinically approved antiviral treatment for ZIKV, the need for the development of potential inhibitors against the virus is essential. In this study, we aimed to screen phytochemicals from papaya ( *Carica papaya L.*) against NS5 protein domains of ZIKV.

**Methods:**

Approximately 193 phytochemicals from an online database (IMPACT) were subjected to molecular docking using AutoDock Vina against the NS5-MTase protein domain (5WXB) and -RdRp domain (5U04).

**Results:**

Our results showed that β-sitosterol, carpaine, violaxanthin, pseudocarpaine, Δ7-avenasterols, Rutin, and cis-β-carotene had the highest binding affinity to both protein domains, with β-sitosterol having the most favorable binding energy. Furthermore, ADMET analysis revealed that selected compounds had good pharmacokinetic properties and were nontoxic.

**Conclusions:**

Our findings suggest that papaya-derived phytochemicals could be potential candidates for developing antiviral drugs against ZIKV. However, further experimental studies using cell lines and *in vivo* models are needed to validate their efficacy and safety.

## Introduction

Zika virus (ZIKV), belonging to the Flaviviridae family, is a mosquito-transmitted virus that infects humans by biting Aedes mosquitos ( *Aedes aegypti*). ^1^ Though the ZIKV was first reported in 1947 in Uganda, the severity of this virus was globally noticed during its outbreak in the years 2015–2017 in Brazil, and later the infection spread to 46 other countries. ^2^^,^^3^ Furthermore, the recent outbreak was associated with severe neurological abnormalities such as microencephaly in foetuses, Guillain-Barré syndrome in adults and newborns due to infected mothers. ^4^^–^^6^ Even though the pandemic waves have subsided, sporadic detections of Zika infections continue to be reported in several parts of the world, with the virus becoming endemic to those regions. ^7^^,^^8^ Henceforth, continuous surveillance and research are essential to develop an effective treatment.

**Figure 1.**
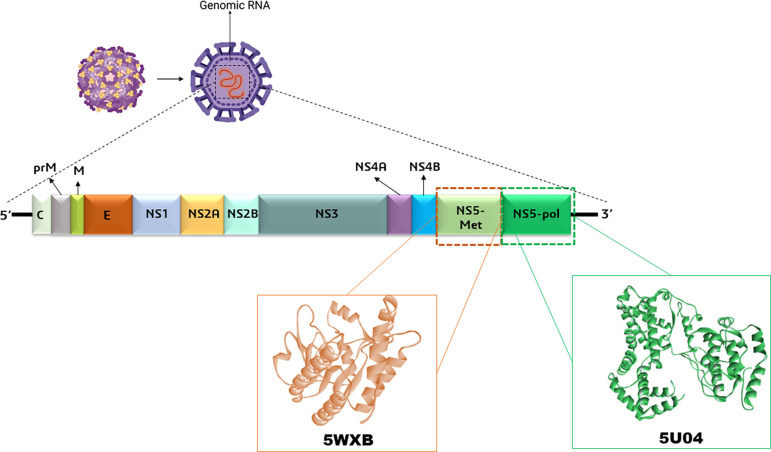
The surface, structural, and non-structural proteins of the ZIKV are illustrated in the diagram, which highlights the virion components and the genomic RNA. The ZIKV polyprotein is composed of seven non-structural proteins (NS1, NS2A, NS2B, NS3 protease and helicase domains, NS4A, NS4B, NS5 methyltransferase, and RNA polymerase domains) and three structural proteins (C, M, and E). In addition, NS5 methyltransferase and RNA polymerase domains’ structures were retrieved from PDB (Protein Data Bank) databases.

ZIKV is an enveloped virus characterized by the presence of a single-stranded RNA genome. ^5^^,^^9^ The genome of ZIKV encodes a single polyprotein (~3400 amino acids), which is translated to three structural proteins (capsid-C, pre-membrane/membrane-prM, and envelope–E) and seven non-structural proteins (NS1, NS2A, NS2B, NS3, NS4A, NS4B, and NS5) using host and viral proteases ( Figure 1). ^10^ Despite the significant efforts of the scientific community, there is currently no specific therapy available for treating ZIKV infection, making developing such antivirals a critical priority. ^11^^,^^12^ Antivirals that could target protein structures involved in genome replication, viral fusion, and RNA synthesis can be highly effective against the ZIKV. Among all the proteins expressed by ZIKV, the most significant and most conserved protein is the non-structural-5 or NS5, which is the polymerase enzyme; it consists of two major domains: RNA methyltransferase (MTase) at its N-terminus and RNA-dependent-RNA polymerase (RdRp) at its C-terminal. ^13^ RdRP is an essential protein domain for initial viral replication, whereas the MTase domain is responsible for RNA capping of the viral genome. The structure of NS5 is exclusive to ZIKV and has no similarity with the host system, which makes it a unique target for inhibitors against ZIKV. ^14^

Continued clinical research is necessary to discover new antivirals. Considering the lower toxicity of plant-derived compounds, ^15^^,^^16^ they serve as promising leads for developing novel antiviral agents against various viruses, including Zika, through various mechanisms involving inhibition of viral replication, modulation of host immune response, and blocking viral entry into host cells. ^17^^–^^21^ Molecular modeling techniques are widely used to study the dynamics, energy, and interactions between biomolecules, including proteins. These techniques are used extensively to study protein-ligand interaction and to predict the drug’s binding mode within the protein’s binding site. Through *in silico* analysis, several studies have identified potential phytochemicals and their impact on human target proteins. ^22^^–^^25^

Henceforth, for this project, we have carried out *in silico* molecular docking analysis for potential anti-ZIKV compounds from *Carica papaya (*commonly referred to as papaya), an edible tropical fruit well-known for its many medicinal properties. ^26^ *C. papaya*, belonging to the family Caricaceae, is a tropical fruit-bearing plant known for its rich content of vitamins, enzymes, and antioxidants. ^27^ Papaya contains bioactive substances, including alkaloids, flavonoids, and phenolic acids, which have been reported to exhibit various pharmacological effects, including antioxidant, anti-inflammatory, immunomodulatory, and antiviral activities. ^27^^,^^28^

*C. papaya* has demonstrated significant therapeutic potential and has been used in home remedies for centuries. ^29^ In several Asian countries, its seeds and peels are utilized to treat stomach ailments, bacterial infections, and inflammations. Moreover, a few studies have reported its antiviral attributes and immunomodulatory properties, but limited research has explored the specific constituents of *C. papaya* that exhibit antiviral effects. Previous *in silico* studies have documented the potential therapeutic effects of papaya in various human diseases, ^30^^–^^32^ including an earlier study on identifying inhibitory compounds (e.g., luteolin) that targeted the dengue virus’s NS2B/NS3 protease (DENV). ^33^^,^^34^ Given that ZIKV and DENV are members of the same family, we proposed to virtually investigate small molecules from papaya with possible targeting ZIKV NS5 protein domains, and to the best of our knowledge, no prior studies have investigated this possibility.

The current study aims to conduct a virtual screening of bioactive molecules from papaya, followed by an ADMET (absorption, distribution, metabolism, elimination, and toxicity) assessment. Through molecular docking analysis using Autodoc Vina, we identified compounds that showed a promising binding affinity with the ZIKV’s NS5 protein domains (MTase and RdRp). Thus, they constitute potential drug targets, and our results may contribute toward developing effective treatments against this public health priority.

## Methods

### Preparation of molecule database and ligand preparation

Phytocompounds of *C. papaya* were selected from the plant database IMPACT and previously published literature (see the *Underlying data,* Supplementary Table S1). ^34^^–^^38^ The ligands’ 3-dimensional (3-D) structures were retrieved from the PubChem database. The ligands underwent a series of adjustments, such as the addition of polar hydrogens, adding charges, and conducting energy minimization using PyRx Virtual Screening Tool software (v-0.8) (RRID:SCR_018548) with the default parameters. ^39^

### Receptor selection and preparation

The crystal structures of the ZIKV proteins were retrieved from the PDB (Protein Data Bank) database. These included the SAH-binding site of the NS5-MTase, and NS5 RNA-dependent RNA polymerase with their PDB entry 5WXB ^40^ and 5U04, ^41^^,^^42^ respectively. Sinefungin and Sofosbuvir were included as the reference inhibitors for the NS5-MTase and NS5 RdRp, respectively. ^14^^,^^43^^–^^45^ In accordance with standard protocol, the protein structures were treated as receptors. At the start of docking, the receptor protein was optimized by removing any unrelated substructure. Then, the side chains in the protein structure were corrected using default settings like adding hydrogens and removing water molecules. The Molprobity server evaluated selected proteins’ stereo-chemical properties and Ramachandran graph. ^46^ Chimera 1.16 (RRID SCR_004097) generated any residues missing in the selected target protein. After removing nonstandard heteroatoms, polar hydrogens and Gasteiger charge were added. Next, the structural aspects of all targets were enhanced using the steepest descent (100 steps) and conjugate gradient algorithms (0 steps) with an Amber force field (Amber Ff14SB). ^46^ The energy-minimized proteins were then converted into *‘pdbqt’* format using AutoDock Tools 1.5.7 (RRID SCR_012746) by AutoDock.

### Ligand and receptor molecular docking

Docking was performed with Autodock Vina, ^47^^,^^48^ as described in a previous study. ^49^ Briefly, the grid box’s dimensions were fixed at XYZ = 30 Å × 30 Å × 30 Å XYZ = 30 Å × 30 Å × 30 Å which was found to be the best size for the default exhaustiveness (= 8), and the ligand binding site was positioned in the middle of the grid box. AutoDock Vina version 1.1.2 (RRID:SCR_011958) was used to calculate each ligand’s binding energy and pose against the selected protein receptors. Each ligand’s best interaction energy scores (kcal/mol) were ranked and plotted against the reference inhibitor. The results obtained are limited to nine binding modes. The log file included a list with increasing binding energies and binding modes. The binding modes were viewed using the BIOVIA Discovery Studio visualizer - v21.1.0.20298 (Dassault Systemes BIOVIA, Discovery Studio, 2021, SanDiego). ^50^

### ADMET and drug-likeness evaluation

The compounds’ molecular properties and drug-like characteristics were assessed using “Lipinski’s Rule of Five” as the basis of analysis. ^51^ First, 19 phytocompounds were analyzed regarding their physicochemical properties, drug-likeness, toxicity, and ADMET properties using ADMETlab 2.0 and SwissADME. In addition, the physicochemical features of compounds, including lipophilicity (log P), solubility (log S), and polar surface area and volume (PSA), were predicted. The mentioned parameters are necessary as they influence how a drug interacts with transport proteins and enzymes involved in drug clearance.

## Results molecular docking

For the molecular docking analysis, around 193 compounds found in papaya were docked against the two domains of ZIKV protein NS5 MTase (5WXB) and RdRp (5U04). ^35^ As positive ligands, Sinefungin against the NS5-MTase and Sofosbuvir against the NS5-RdRp were docked. Sinefungin had a binding affinity of -8.1 kcal/mol, whereas the binding affinity for Sofosbuvir was -7.4 kcal/mol.

**Figure 2.**
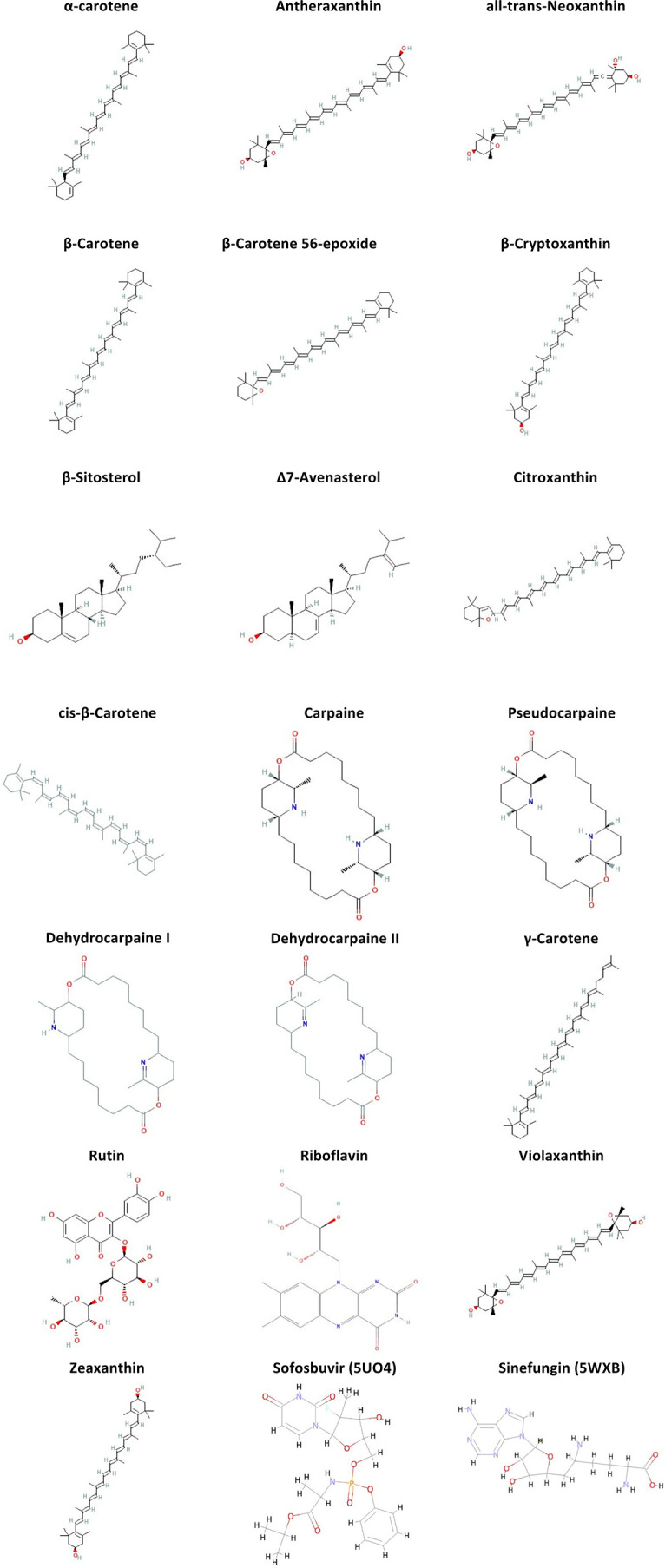
Structural representation (2D) of the ligands shortlisted for having greater binding affinity to the receptor than the positive ligands used (Sofosbuvir and Sinefungin for 5U04 and 5WXB, respectively).

**Figure 3.**
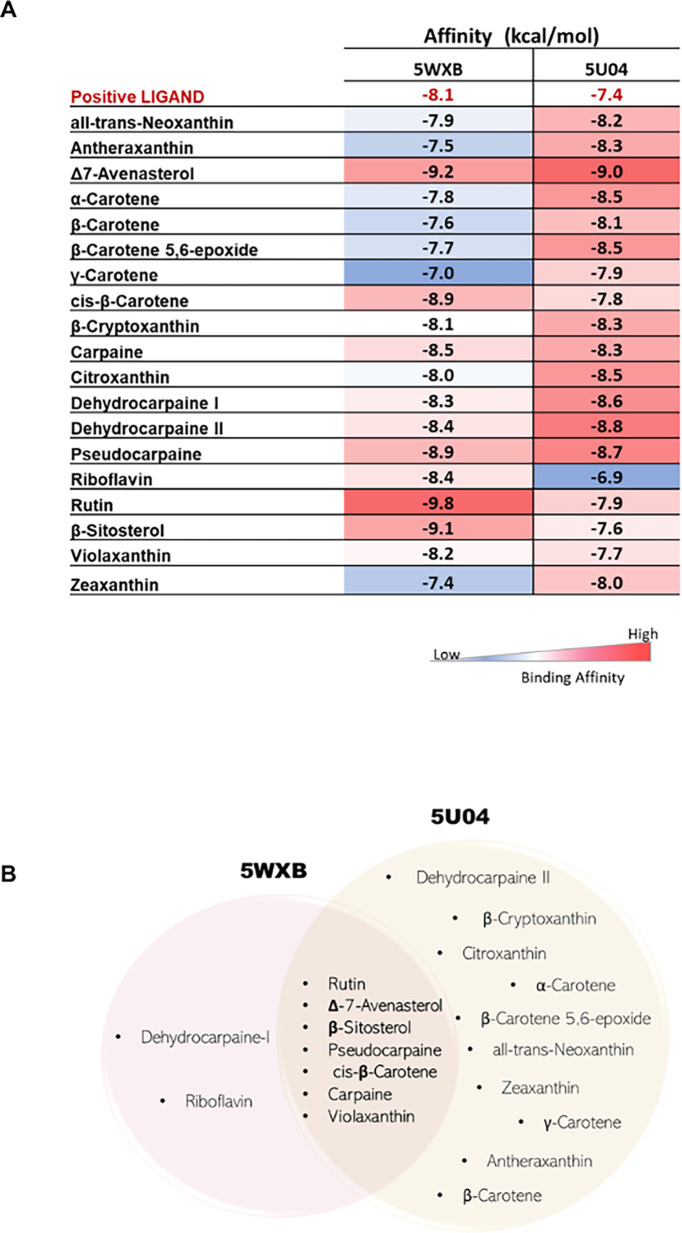
Docking results of phytocompounds from *C. papaya* against target NS5 protein domains NS5-MTase (5WXB) and NS5-RdRp (5U04) of Zika virus (A) The Heatmap showing the binding affinities of best-docked compounds with target protein domains. Blue indicates low binding affinity and red indicates high binding affinity. (B) Venn diagram representing the commonly shared best-docked ligands (compared to respective positive control ligands) with the target protein domains.

Binding affinity is an essential preliminary parameter for assessing a potential candidate drug. Therefore, first of all, we assessed the binding affinity of the candidate drug and compared it with the positive controls (Sinefungin and Sofosbuvir against 5WXB and 5U04, respectively). The initial docking analysis helped narrow it down to 19 shortlisted compounds that showed higher affinity than their respective positive ligands, as shown in Figure 2. Figure 3A shows the heat map of the binding affinity of the ligands, with the lowest energy/highest affinity corresponding to red color, whereas the blue color indicates the highest binding energy or/lowest affinity.

**Figure 4.**
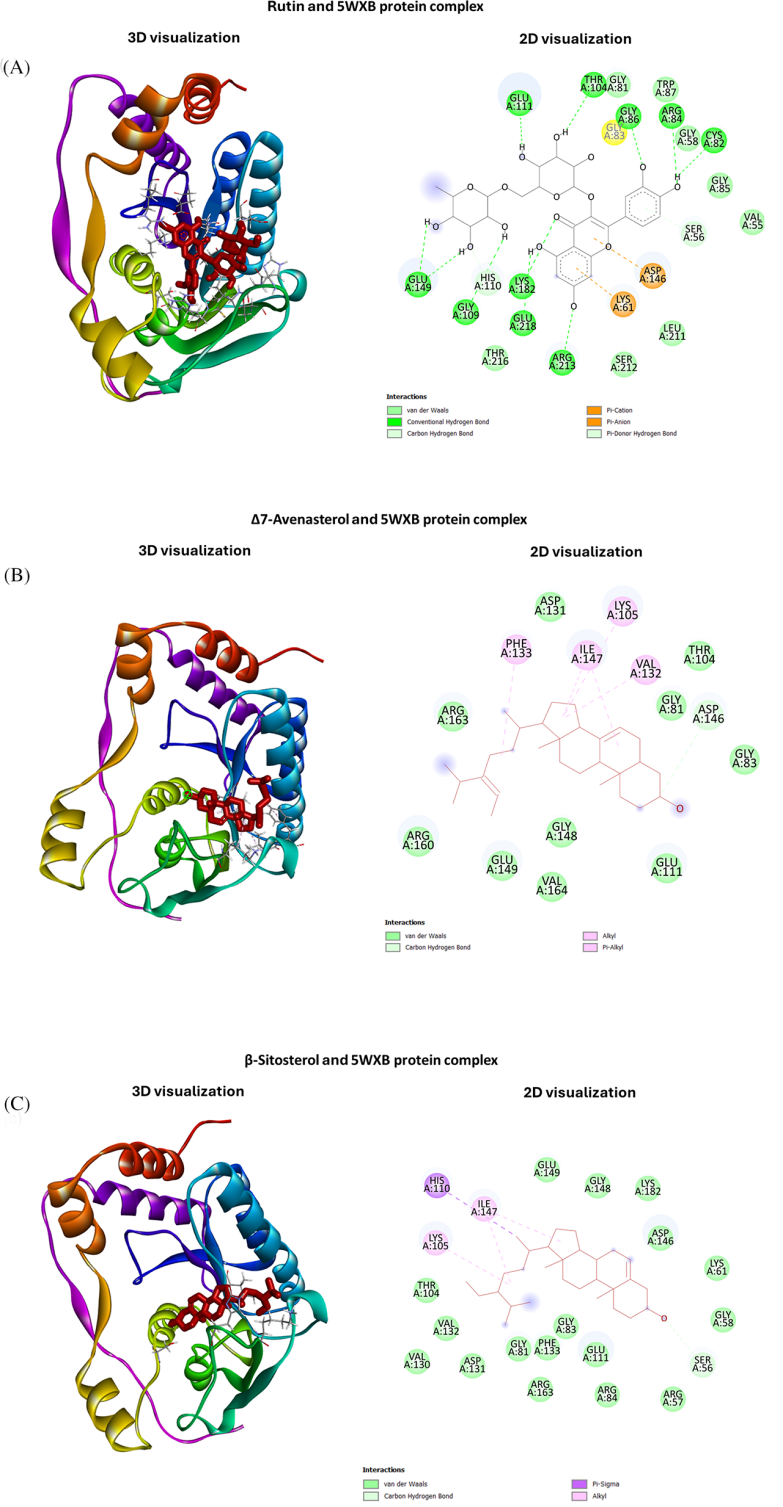

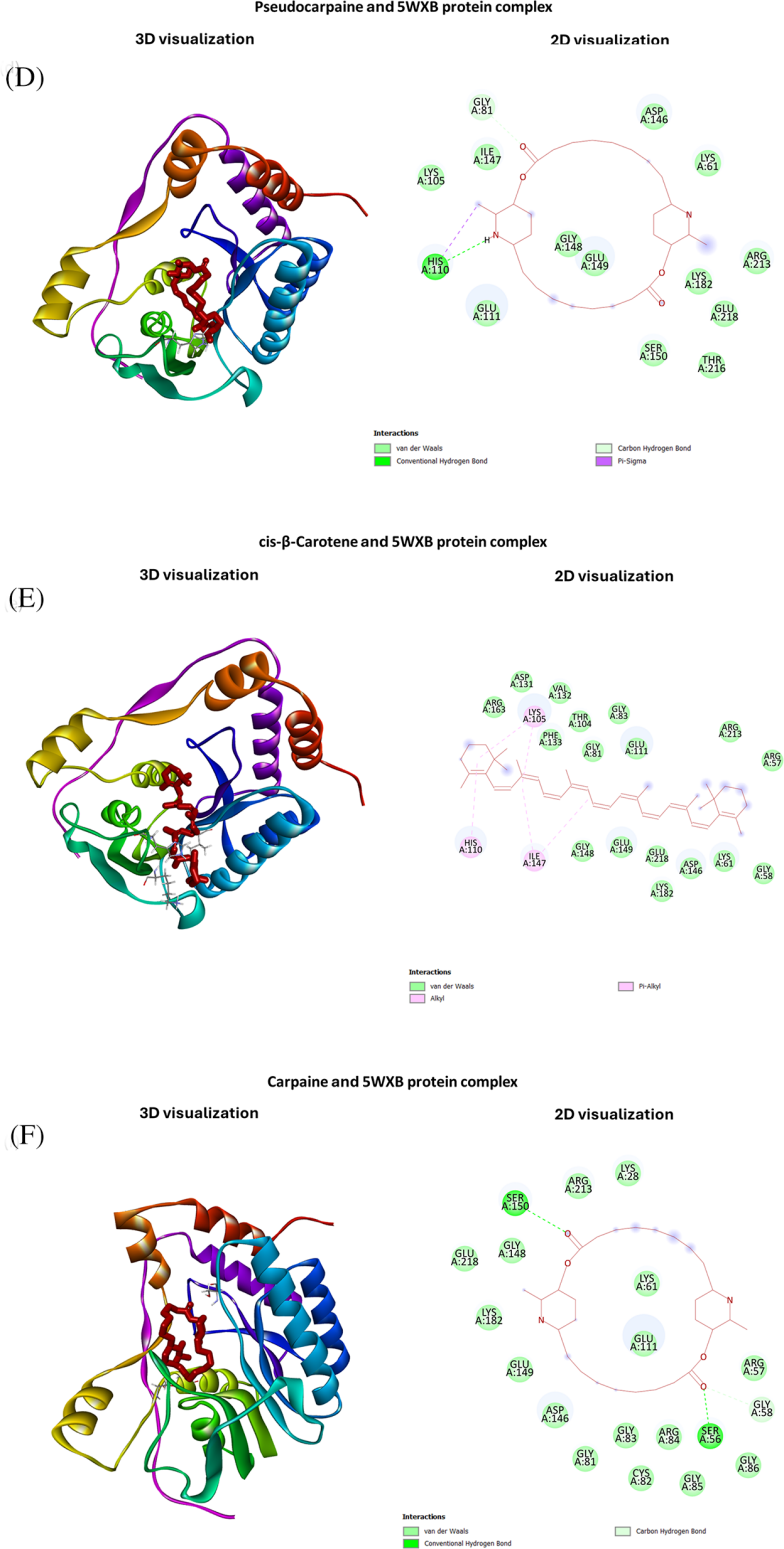

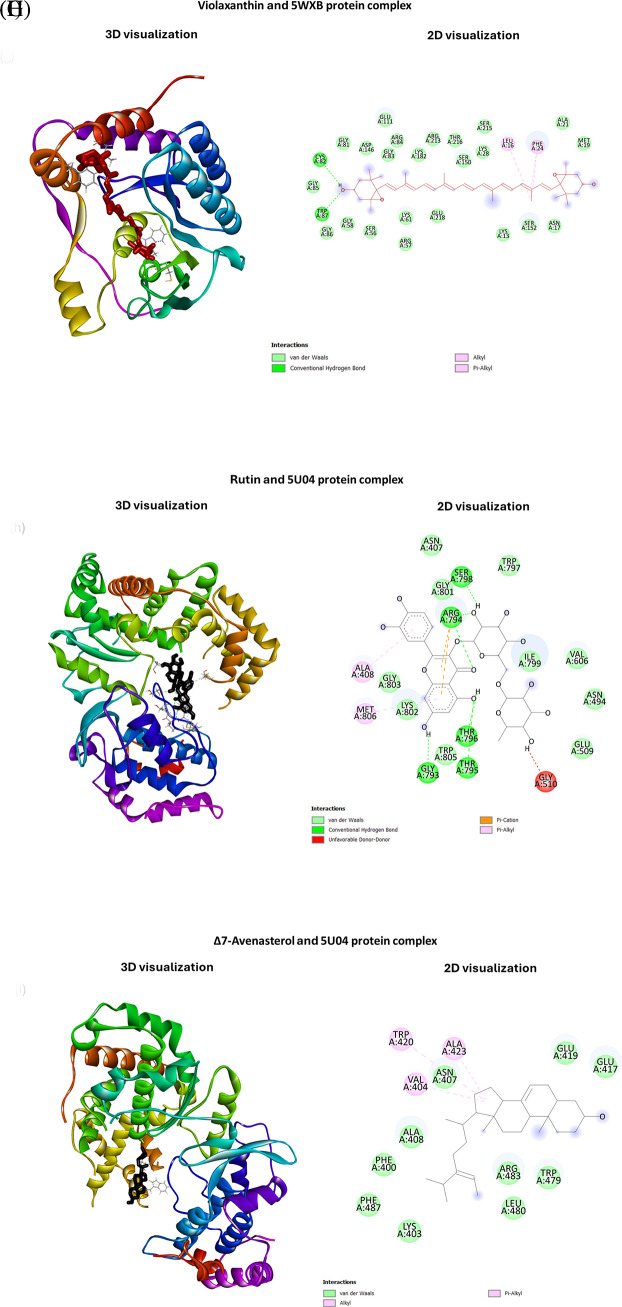

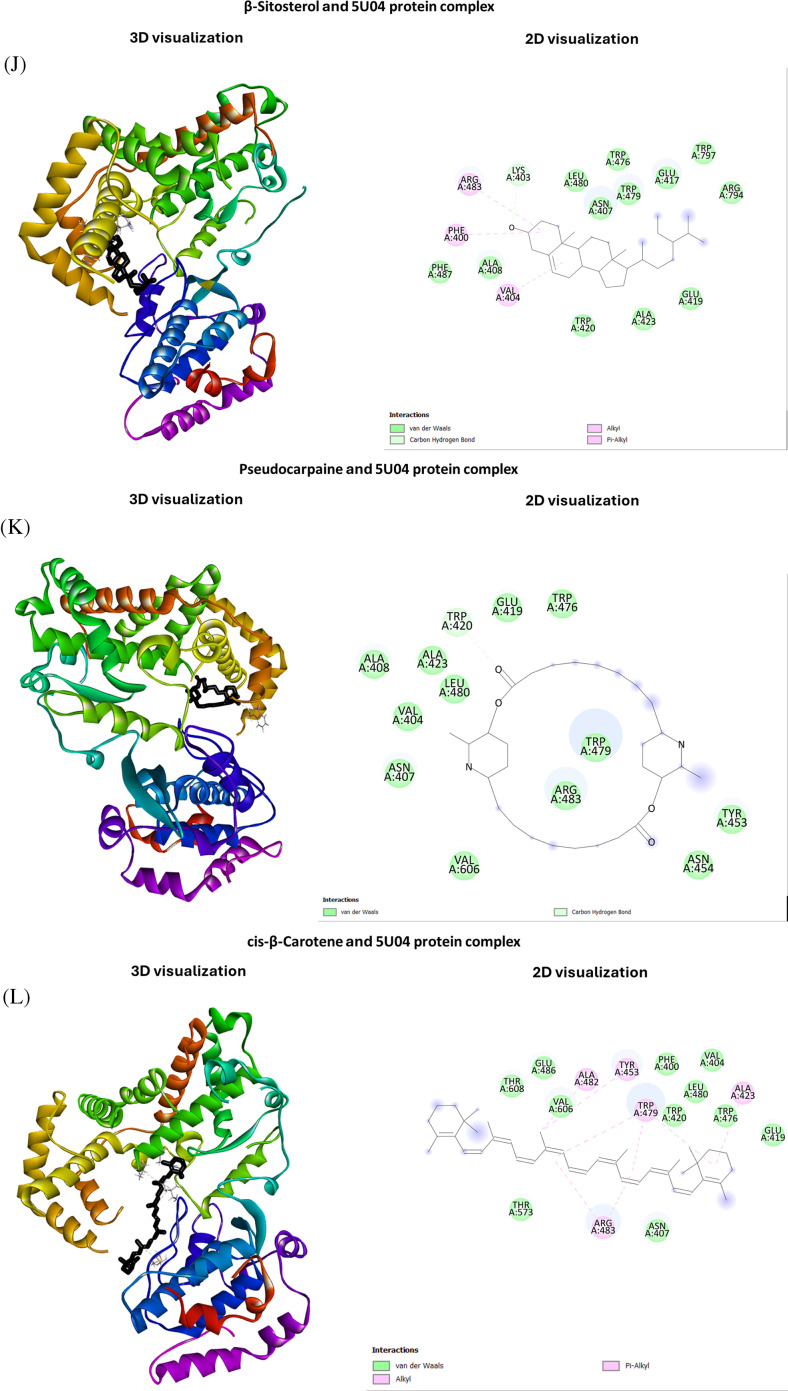

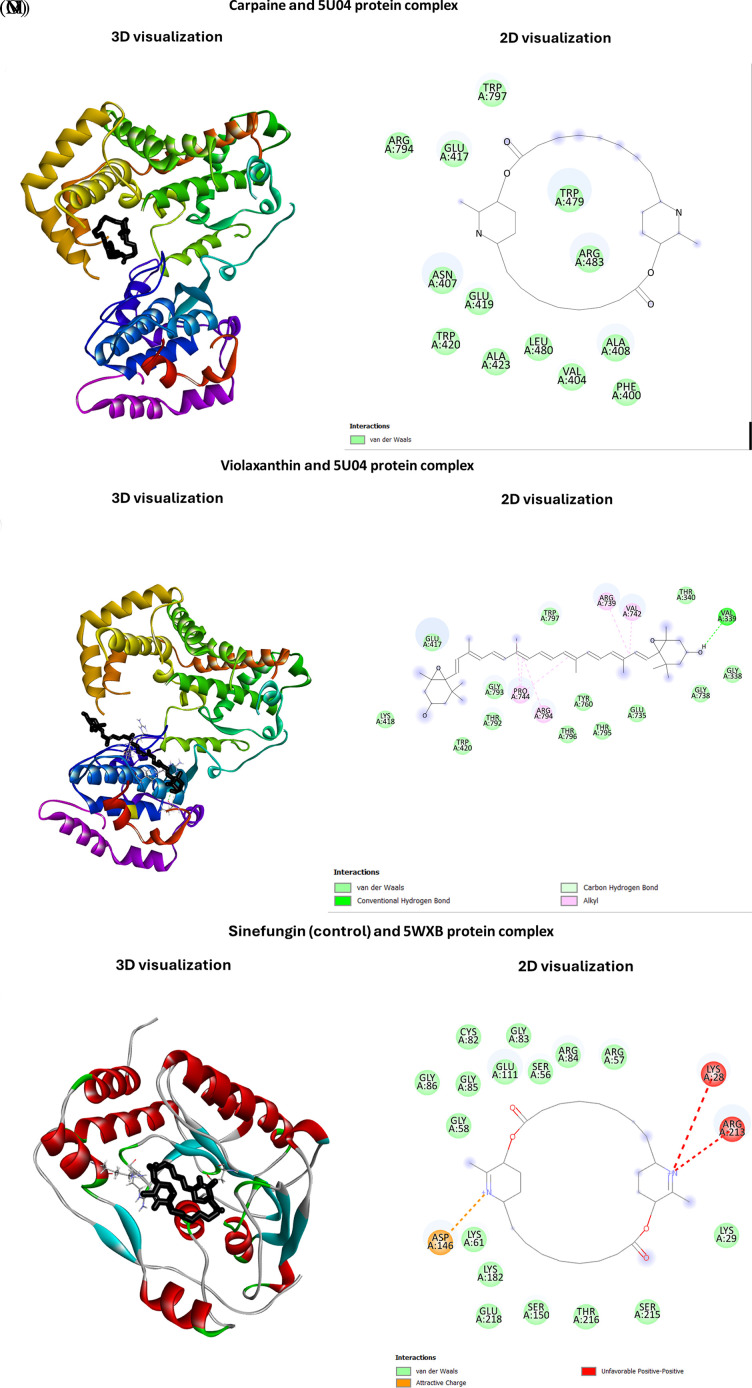

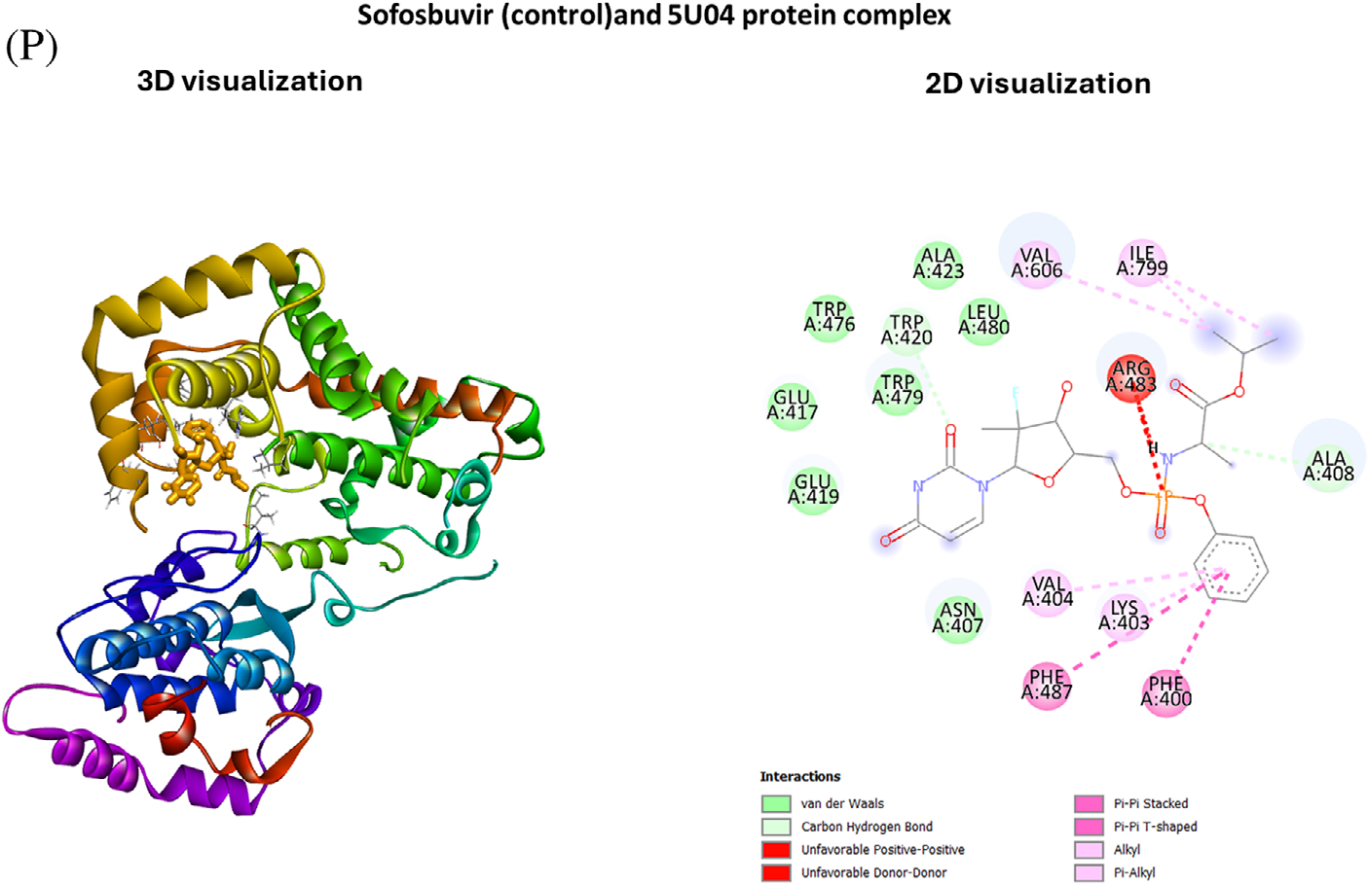
2D and 3D interaction views of the best-docked phytocompounds from *C. papaya*, illustrating their binding modes with ZIKV NS5 protein domains. (A)-(G) display the complexes with MTase-5WXB, (H)-(N) present the complexes with RdRp-5U04, and (O)-(P) show the complexes with positive controls sinefungin and sofosbuvir.

For the MTase domain (5WXB), rutin has the strongest binding affinity with a binding energy of -9.80 kcal/mol, followed by Δ7-avenasterol (-9.20 kcal/mol), β-sitosterol (-9.10 kcal/mol), cis-β-carotene (-8.90 kcal/mol) and pseudocarpaine (-8.90 kcal/mol). On the other hand, the weakest binding affinity is shown by γ-carotene, with a value of -7.00 kcal/mol. Whereas for the RdRp domain of NS5 (5U04), the ligand Δ7-avenasterol shows the highest binding affinity, followed by dehydrocarpaine-II and pseudocarpaine. Several ligands (rutin, carpaine, Δ7-avenasterol, β-sitosterol, pseudocarpaine, cis-β-carotene, violaxanthin) showed a better binding affinity with both the domains of NS5- protein ( Figure 3B). Figure 4 shows the 3D and 2D structure of the common ligands, which showed higher docking ability towards both the domains of NS5 protein; however, the remaining ligand complexes are mentioned in the *Underlying data,* Figures S1 and S2. ^35^ Among these bispecific compounds, rutin showed the highest binding affinity for both protein domains, followed by carpaine.

Hydrogen bonds and hydrophobic interactions play a critical role in molecular docking, and these interactions are key components in identifying potential drugs. As mentioned in Table 1, rutin forms the highest number of hydrogen bonds involving 14 amino acid residues (ARG84, GLY86, LYS182, ARG213, CYS82, GLU218, GLU149, GLU149, GLY109, THR104, GLU111, HIS110, HIS110, LYS61) and the bonding distance ranging from 2.27 to 3.08 Å; however, rutin had no hydrophobic interaction with the MTase domain ( Figure 4). In hydrophobic interactions, Δ7-Avenasterol and cis-β-Carotene form the highest number (5) bonds with the MTase domain. Ligands interaction with the RdRp domain of NS5 ( Table 2, Figure 4) shows that rutin has formed the highest number of hydrogen bonds (ARG794, ARG794, THR795, THR796, GLY793, SER798) and three hydrophobic interactions. α-Carotene forms the highest number of hydrophobic interactions with the RdRp domain and has no hydrogen bonding.

**Table 1.** Docking results of identified potential phytochemical compounds from *C. papaya* against NS5- MTase domain (5WXB).

Ligand name	Hydrogen bond			Hydrophobic bond		
	Number	Amino acids involved	Distance (Å)	Number	Amino acid	Type
Sinefungin (Positive Ligand)	5	ARG84, GLY85, GLU111, THR104, VAL130	2.03, 2.43, 2.16, 2.12, 2.52	0	**-**	**-**
Rutin	14	ARG84, ARG213, GLY86, LYS182, CYS82, GLU218, GLU149, GLU149, GLY109, THR104, GLU111, HIS110, HIS110, LYS61	2.76, 2.55, 3.08, 2.27, 2.33, 2.34, 2.73, 2.62, 2.96, 2.64, 2.90, 2.57, 2.61, 2.81	0	**-**	**-**
Riboflavin	9	SER56, GLY86, TRP87, GLY81, GLU111, GLY58, CYS82, GLY86, ASP146	2.20, 2.56, 2.67, 2.55, 2.60, 2.34, 2.39, 2.26, 3.66	0	**-**	**-**
Carpaine	3	SER56, SER150, GLY58	1.98, 2.81, 2.90	0	**-**	**-**
Pseudocarpaine	3	HIS110, GLY81, GLY812	2.57, 2.54, 2.63	1	HIS110	Pi-Sigma
Violaxanthin	2	TRP87, CYS82	2.47, 2.61	3	LEU16, PHE24, PHE24	Alkyl, Pi-Alkyl, Pi-Alkyl
Dehydrocarpaine I	2	GLU149, SER150	4.86, 2.71	0	**-**	**-**
β-Sitosterol	1	SER56	2.81	4	HIS110, LYS105, ILE147, ILE147	Pi-Sigma, Alkyl, Alkyl, Alkyl
Δ7-Avenasterol	1	ASP146	3.56	5	LYS105, VAL132, ILE147, ILE147, PHE133	Alkyl, Alkyl, Alkyl, Alkyl, Pi-Alkyl
cis-β-Carotene	0	**-**	**-**	5	LYS105, LYS105, ILE147, ILE147, HIS110	Alkyl, Alkyl, Alkyl, Alkyl, Pi-Alkyl

**Table 2.** Docking results of identified potential phytochemical compounds from *C. papaya* against NS5-RdRp domain (5U04).

Ligand name	Hydrogen bond			Hydrophobic bond		
	Number	Amino acids	Distance (Å)	Number	Amino acids	Type
Sofosbuvir (Positive ligand)	2	TRP420, ALA408	3.05, 3.43	-	-	-
Rutin	6	ARG794, ARG794, THR795, THR796, GLY793, SER798	2.24, 2.36, 3.05, 2.76, 2.06, 2.28	3	ARG794, MET806, ALA408	Pi-Alkyl, Pi-Alkyl, Pi-Alkyl
Zeaxanthin	3	ASN612, ASP665, GLY664	2.12, 2.15, 2.55	3	VAL606, ILE799, MET806	Alkyl, Alkyl, Alkyl
Violaxanthin	2	VAL339, VAL339	2.42, 2.99	6	ARG739, VAL742, PRO744, PRO744, ARG794, PRO744	Alkyl, Alkyl, Alkyl, Alkyl, Alkyl, Alkyl
β-Sitosterol	1	LYS403	2.44	3	VAL404, ARG483, PHE400	Alkyl, Alkyl, Pi-Alkyl
β-Carotene-5,6-epoxide	1	GLY738	3.04	7	ALA423, ARG739, VAL742, ARG794, ARG794, TRP797, TRP797	Alkyl, Alkyl, Alkyl, Alkyl, Alkyl, Pi-Alkyl, Pi-Alkyl
Citroxanthin	1	GLY738	3.04	7	ALA423, ARG739, VAL742, ARG794, ARG794, TRP797, TRP797	Alkyl, Alkyl, Alkyl, Alkyl, Alkyl, Pi-Alkyl, Pi-Alkyl
All-trans-Neoxanthin	1	ASN407	2.22	7	ARG739, VAL742, PRO744, RG794, ARG794, TRP797, TRP797	Alkyl, Alkyl, Alkyl, Alkyl, Alkyl, Pi-Alkyl, Pi-Alkyl
Pseudocarpaine	1	TRP420	2.42	0	**-**	**-**
Dehydrocarpaine II	1	ALA408	2.37	0		
Carpaine	-	-	-	-	-	-
γ-Carotene	0	**-**	**-**	13	TRP797, ALA423, LEU480, ARG739, AL742, VAL742, PRO744, ARG794, ARG794, VAL404, TRP479, TRP797, TRP797	Pi-Sigma, Alkyl, Alkyl, Alkyl, Alkyl, Alkyl, Alkyl, Alkyl, Alkyl, Alkyl, Pi-Alkyl, Pi-Alkyl, Pi-Alkyl
cis-β-Carotene	0	**-**	**-**	8	ALA423, LA482, ARG483, RG483, TYR453, TRP479, TRP479, TRP479	Alkyl, Alkyl, Alkyl, Alkyl, Pi-Alkyl, Pi-Alkyl, Pi-Alkyl, Pi-Alkyl
β-Carotene	0	VAL606	5.12	5	VAL606, ILE799, ILE799, MET806, TYR609	Alkyl, Alkyl, Alkyl, Alkyl, Pi-Alkyl
Antheraxanthin	0	ARG739	3.99	6	ARG739, VAL742, ARG794, ARG794, TRP797, TRP 797	Alkyl, Alkyl, Alkyl, Alkyl, Pi-Alkyl, Pi-Alkyl
β-Cryptoxanthin	0	**-**	**-**	5	ALA423, ARG739, VAL742, PRO744, TRP797	Alkyl, Alkyl, Alkyl, Alkyl, Pi-Alkyl
α-Carotene	0	**-**	**-**	11	TRP420, TRP420, LYS421, ARG739, VAL742, ARG794, ARG794, TRP420, TRP420, TRP420, RP797	Pi-Sigma, Pi-Sigma, Alkyl, Alkyl, Alkyl, Alkyl, Alkyl, Pi-Alkyl, Pi-Alkyl, Pi-Alkyl, Pi-Alkyl
7-Avenasterol	0	**-**	**-**	3	VAL404, ALA423, TRP420	Alkyl, Alkyl, Pi-Alkyl

### Prediction of ADMET analysis

ADMET and drug-likeness evaluation provide insight into the properties of drugs based on their chemical structure. The best-docked ligands ( Figure 2) were analyzed for their pharmacokinetic properties. The evaluation criteria were based on solubility, gastrointestinal absorption (GI), blood-brain barrier (BBB) permeability, and violation of Lipinski’s rules.

(BBB - permeability = YES or > 0.1); (GI - absorption = high); (Carcinogenicity = 0 to 0.3); (PAINS alert = 0) and (Lipinski’s violation = 0).

All the compounds mentioned in Figure 2 were screened through ADMET for their suitability as a drug. The screening through ADMETLab 2.0 revealed that several compounds were approved under Lipinski criteria. BBB values reflect the ability of the ligands to cross the brain barrier, with a higher score value indicating greater permeability. β-Sitosterol has the highest BBB penetration with a score of 0.84, suggesting effective crossing. Carpaine showed to have poor penetration with a BBB score of 0.01. BBB values ranged between 0.01 and 0.001, indicates poor barrier crossing. Riboflavin, rutin, and all-trans-neoxanthin displayed moderate BBB permeability values ranging from 0.111 to 0.444, while several had BBB penetrations below 0.1, suggesting the limited ability to cross the BBB (see the *Underlying data,* supplementary Table S2. ^35^

Table S2 ( *Underlying data* ^35^ also signifies the ligands’ permeability based on Caco-2 cell permeability values, which translates to intestinal epithelium permeability. For example, β-sitosterol had the highest Caco-2 cell permeability value at -4.756, signifying improved intestinal permeability. On the other hand, carpaine showed poor intestinal epithelium permeability of -4.985. Table S2 ( *Underlying data* ^35^) suggests ligands and their respective Lipinski’s rule of five compliance. Among these ligands, β-sitosterol, violaxanthin, riboflavin, all-trans-neoxanthin, dehydrocarpaine-I, dehydrocarpaine-II, and β-carotene are Lipinski’s-rule-of-five compliant and are considered to be drug-like compounds.

## Discussion

Emerging viruses such as Dengue, Zika, Ebola, SARS-CoV2, and other infectious viruses demonstrate that the current antiviral therapeutic regimen is insufficient for these pathogens. ^52^ The coronavirus 2019 (COVID-19) pandemic further highlighted this inadequacy. Vaccine development is a time-consuming and lengthy process, which also faces the challenges of large-scale administration. Moreover, the faster-evolving attribute of RNA viruses also makes it challenging to develop a particular antiviral treatment, ^53^ one such RNA virus, Zika, remains a global concern. Zika-related disorders are mainly found in infants but can also affect adults. Zika-related disorders reported in adults were the cases of Guillain Barre’ Syndrome, ^54^ Myelitis, ^55^ Meningoencephalitis, ^56^ and Uveitis. ^57^ Currently, no approved drugs and no vaccines are available for treating ZIKV infection.

Plant-derived natural compounds are promising alternatives for treating infections with minimal side effects. Medicinal plants are the richest source of new drugs, including antivirals targeting several human ailments. Previous clinical studies have demonstrated the successful inhibition of Dengue infection by papaya extract. ^58^^,^^59^ Phytochemical screening of papaya has been reported to constitute several compounds, which have potent therapeutic effects against several human diseases, such as inflammation, oxidative stress, antiviral and hypoglycemia. ^60^^,^^61^ Since Zika and Dengue belong to the same family of viruses, we hypothesized that papaya could also serve as the source for identifying potential inhibitors against ZIKV infection. In this context, in the present study, we have performed molecular docking to identify possible lead compounds from papaya against NS5 protein domains of the ZIKV.

Initially, we screened 193 papaya-derived phytochemicals to know their molecular docking potential with the NS5 protein domains of the ZIKV. Our analysis revealed seven compounds (β-sitosterols, carpaine, violaxanthin, rutin, β-carotene, pseudocarpaine, and Δ7-avenasterol) that exhibited bispecific docking activity against both NS5 domains, with higher docking scores compared to their respective positive ligands (Sinefungin and Sofosbuvir against 5WXB and 5U04, respectively) ( Figure 3A). The MTase activity of the ZIKV NS5 protein plays a crucial role in the replication and spread of the virus. ^62^ Hence, inhibiting this activity can be an effective strategy to prevent the virus from spreading. Previous studies explored small molecule inhibitors and RNA-based inhibitors against ZIKV infections. For instance, Sinefungin is a small molecule that has been shown to inhibit the MTase activity of several flaviviruses. ^63^ However, the study showed a low potency of Sinefungin on NS5 of ZIKV compared to other flaviviruses’ MTase domain; additionally, the toxicity associated with the particular drug had raised concern about its use. ^64^

Hydrogen bonding and hydrophobic interactions are crucial in facilitating significant ligand binding at the active site residues of the receptor in docked complexes. ^65^ In addition to hydrogen bonds and hydrophobic interactions, other non-covalent interactions such as alkyl-alkyl, pi-alkyl, and pi-pi interactions also play significant roles in ligand binding. Alkyl-alkyl interactions stabilize the complex via van der Waals forces, while pi-alkyl and pi-pi interactions enhance stability and binding affinity through aromatic ring stacking and hydrophobic effects. However, we primarily focused on hydrogen bonds and hydrophobic interactions due to their critical roles in ligand binding affinity and stability within the receptor's active site. ^65^^,^^66^ The bioactive compounds in this study were found to form hydrogen bonds and hydrophobic interactions with MTase. In the present study, flavonoid compounds, like rutin, showed the highest binding affinity to the NS5-MTase domain (5WXB), followed by riboflavin. Rutin formed a complex that entirely occupied the protein through 14 hydrogen bonding interactions with no hydrophobic interactions, and riboflavin interacted with the NS5-MTase through nine hydrogen bonds ( Table 1, Figure 4). Moreover, the interacting residues were identified as essential residues of the substrate binding of the MTase. ^67^ The observed hydrogen bonding interactions between the selected compounds from papaya and MTase suggest that these compounds may effectively occupy the substrate binding site of MTase, making them promising lead compounds for drug development against ZIKV infection. Furthermore, the present study also indicates that phytocompounds from papaya showed several forms of strong and stable bonds like carbon-hydrogen, pi-alkyl, van der Waals, covalent, hydrophobic, and electrostatic bonds with the receptor ligands ( Tables 1 and 2).

The ZIKV RdRp constitutes another important target for inhibiting Zika viral replication. ^68^ ZIKV RdRp consists of two binding sites, the first one being the active site (formed by Asp535, Trp797, and Ile799), while the other side is the allosteric or N pocket, which contains the priming loop that is essential for stabilizing the initiation complex and releasing new dsRNA. ^69^ In this study for docking to the RdRp domain (5U04), the result reveals that rutin, has the highest number of hydrogen bond interactions (with ARG794, ARG794, THR795, THR796, GLY793, SER798) and hydrophobic interactions (involving ARG794, MET806, ALA408 amino acids) compared to the positive ligand, Sofosbuvir ( Table 2, Figure 4).

Phytosterols are naturally occurring plant molecules with a structure similar to cholesterol, ^70^ and they have been demonstrated to possess the antiviral activity of several sterols against the spike protein of the COVID-19 virus and influenza-A virus. ^63^^,^^71^ Our study identified several phytosterols, including β-sitosterol and Δ7-avenasterol, demonstrating potent inhibitory affinity ( Figure 3A). Earlier studies have also shown the potential health benefits, including antiviral effects of β-sitosterol, which has been shown to reduce the infectivity of the hepatitis-B virus and HIV, possibly by hindering the attachment of viruses to host cells. ^72^ Another study on HIV patients found that the combination of β-sitosterol and β-sitosterol glycoside helped maintain stable CD4 cell counts and significantly reduced plasma viral loads. ^73^

Alkaloids have also been shown to inhibit DNA and/or RNA synthesis in multiple viruses, including human coronavirus and herpes simplex virus. ^74^^–^^76^ Our docking studies identified alkaloids like carpaine and pseudocarpaine ( Figure 3A) with higher binding affinity to both target protein domains compared to positive ligands. Besides, another alkaloid dehydrocarpaine-II had a good binding score (-8.8 kcal/mol) only towards the protein NS5- RdRp domain. Therefore, binding to the RdRp domain may help inhibit normal virus replication. Meanwhile, the carpaine-5U04 complex had no interaction with the protein through hydrogen or hydrophobic bonding.

Apart from it, several carotenoids had also shown good binding affinity to both domains of NS-5. Especially, carotenoids such as α-carotene, β-carotene, citroxanthin, β-cryptoxanthin, γ-carotene, violaxantin, and zeaxanthin showed higher binding affinity to the NS5-RdRp than NS5-MTase. Carotenoids have a variety of applications, including anticancer, anti-inflammatory, and antioxidant properties, as well as anti-obesity. ^77^^–^^79^ Moreover, carotenoids also possess antiviral properties. ^80^^–^^82^ Also, recent studies have proven the antiviral properties of carotenoids against COVID-19. ^83^

Conducting *in silico* analyses on compounds to assess their absorption, distribution, metabolism, and excretion (ADME) properties is critical as part of drug development. ^84^ Failure to accurately simulate these attributes or assess any toxicities may cause inhibitors to fail the screening process and thus fall outside its criteria for approval. ^85^ BBB penetration is important when developing drugs to treat central nervous system (CNS) conditions. ^86^ Cytochrome P450 (CYP) isozymes metabolize drugs, fatty acids, steroids, bile acids, and carcinogens. ^87^ Approximately 75% of phase-1 drug metabolism processes involve CYP enzymes. ^88^ CYP inhibitor and substrate scores were calculated in this metabolism, and the result shows that the shortlisted compounds are non-substrates and non-inhibitors of CYP enzymes ( *Underlying data*, supplementary Table S2 ^35^). Our ADMET analysis ( *Underlying data*, supplementary Table S2 ^35^) demonstrated that 19 of the best-docked compounds ( Figure 2) had nontoxic properties. Lipinski’s rule of five was violated for ten compounds ( *Underlying data*, supplementary Table S2 ^35^). However, the remaining compounds (carpaine, dehydrocarpaine I, dehydrocarpaine II, pseudocarpaine, Δ7-avenasterol, all-trans-neoxanthin, riboflavin, β-sitosterol, and violaxanthin) were found to be acceptable candidates based on Lipinski’s rule of five.

Zika virus infection remains a public health concern due to the lack of specific antiviral therapies. Our study investigated the potential of *C. papaya* extracts as a source of ZIKV inhibitors. *In silico* molecular docking identified seven compounds with favorable binding energies to both the MTase and RdRp domains of the ZIKV NS5. These findings suggest their potential as the inhibitors of viral replication. However *, in silico* methods require *in vitro* and *in vivo* validation to confirm their efficacy against ZIKV infection. Furthermore, these compounds' bioavailability and toxicity profiles need to be assessed to ensure their safety and effectiveness as potential drug candidates. These results offer a promising approach for developing natural, safe, and effective antiviral drugs against ZIKV, potentially filling the current therapeutic gap and contributing to global health efforts against the virus.

## Data Availability

Protein Data Bank: crystal structure of ZIKV MTase in complex with SAH. Accession number 5WXB; https://doi.org/10.2210/pdb5WXB/pdb . Protein Data Bank: Crystal structure of Zika virus NS5 RNA-dependent RNA polymerase https://doi.org/10.2210/pdb5U04/pdb. Zenodo: In silico screening for potential inhibitors from the phytocompounds of Carica papaya against Zika virus NS5 protein. https://doi.org/10.5281/zenodo.12057456 . ^35^ This project contains the following underlying data:
• 3D structure-Papaya compounds-IMPACT.zip (3D structures of all the compounds downloaded from IMPACT database) • NS5- protein.zip (3D structures of both the protein domains of NS5 protein) • supplementary Figures.docx • Supplementary Table S1.xlsx (Molecular docking result of all the downloaded compounds) • supplementary Table S2.xlsx (ADMETlab 2.0 information of the shortlisted compounds) Data are available under the terms of the Creative Commons Attribution 4.0 International license (CC-BY 4.0).
