# Supplementary material for: In silico screening for potential inhibitors from the phytocompounds of Carica papaya against Zika virus NS5 protein
Source: F1000Res. 2024 Jun 25;12:655. Originally published 2023 Jun 13. [Version 2] doi: 10.12688/f1000research.134956.2 (PMC11310656; doi:10.12688/f1000research.134956.2)
Supplement: Peer review history

# Reviewer report: Reviewer response for version 2

**DOI:** 10.5256/f1000research.168075.r295619

**Published:** 2024-08-08

**Authors:** Luis Marquez-Dominguez

**Affiliations:** 1 Instituto Mexicano del Seguro Social, Puebla, Mexico

The authors have made several corrections. However, in this revision, I have some comments or doubts that could better enrich the authors' work.

1. **Methods**: The plant database is IMPPAT: Indian Medicinal Plants, Phytochemistry And Therapeutics, not IMPACT.
2. **Molecular Docking Results**:
  - **Second Paragraph**: The text "Figure 3A shows the heat map of the binding affinity of the ligands, with the lowest energy/highest affinity corresponding to red color, whereas the blue color indicates the highest binding energy or/lowest affinity." should be changed to "Figure 3A shows the heat map of the binding affinity of the ligands. The red color represents the lowest energy/highest affinity, while the blue color indicates the highest energy/lowest affinity."
3. **Page 5, Last Paragraph**: The statement "rutin forms the highest number of hydrogen bonds involving 14 amino acid residues" is incorrect. Rutin interacts with 12 residues but generates two hydrogen bonds with two residues, Glu149 and His110. It is suggested to correct this. If the author wants to emphasize that there are two hydrogen bonds, they could indicate this by writing "2-E149," where the number corresponds to the hydrogen bonds formed with the residue. It is also recommended to list the amino acids in ascending order and use the one-letter nomenclature for the amino acids.
4. **Figure 3**:
  - Order the red molecules at the top and the blue molecules at the bottom. This will allow a quick evaluation of which molecules are potential inhibitors.
  - In the column titles, put the name or function of the enzyme and leave the PDB ID in parentheses.
5. **Figure 4**:
  - The control should be the first to appear, as it is the reference for comparison.
  - Reduce the number of structures presented. Select only two for each protein and two that inhibit both. For example, for 5WXB: Rutin and delta 7 avenasterol; for 5U04: delta 7 avenasterol and dehydrocarpaine. The other couplings can be included in the supplementary material.
  - If possible, all structures should present the same pose to facilitate comparison of inhibitors for the same enzyme.
6. **Table 1**:
  - Modify the amino acid nomenclature to one letter.
  - Be careful with the number of amino acids. For example, pseudocarpaine lists Gly812, but 5WXB has only 195 amino acids.
  - Remove the column title "Hydrophobic bond" and replace it with "Non-covalent interactions."
7. **Table 2**: Similar suggestions to Table 1.
  - Mention in the text that the crystallized protein 5U04, according to the PDB sequence, has 598 amino acids, but parts of loops 1 (residues 340–363), 3 (residues 408–417), 4 (residues 454–471), 5 (residues 533–542), and 6 (residues 576–606) are missing in the model. This is in the reference for the crystallization of the model.
  - Review the amino acid numbering; for example, gamma-carotene lists AL742, and cis-beta-carotene lists LA482 and RG483, alpha-carotene lists RP797, which are incorrect.
8. **Page 16**: The section "(BBB - permeability = YES or > 0.1); (GI - absorption = high); (Carcinogenicity = 0 to 0.3); (PAINS alert = 0) and (Lipinski’s violation = 0)" is disjointed from the text. Integrate it into the following paragraph, mentioning that these are the reference values for these variables.

These would be my recommendations.

Is the work clearly and accurately presented and does it cite the current literature?

Partly

If applicable, is the statistical analysis and its interpretation appropriate?

Not applicable

Are all the source data underlying the results available to ensure full reproducibility?

Partly

Is the study design appropriate and is the work technically sound?

Partly

Are the conclusions drawn adequately supported by the results?

Partly

Are sufficient details of methods and analysis provided to allow replication by others?

Partly

Reviewer Expertise:

virology, antiviral design, rational drug design, and enzyme kinetics, molecular biology.

I confirm that I have read this submission and believe that I have an appropriate level of expertise to confirm that it is of an acceptable scientific standard.

# Reviewer report: Reviewer response for version 2

**DOI:** 10.5256/f1000research.168075.r295618

**Published:** 2024-07-17

**Authors:** Arumugam Vijaya Anand

**Affiliations:** 1 Department of Human Genetics and Molecular Biology, Bharathiar University, Coimbatore, India

1. This suggestion is not properly carried out and it should be rectified. The use of abbreviations in the abstract (ADME) section may distract readers who wish to quickly skim through several publications before deciding to read one in full.

Is the work clearly and accurately presented and does it cite the current literature?

Yes

If applicable, is the statistical analysis and its interpretation appropriate?

Not applicable

Are all the source data underlying the results available to ensure full reproducibility?

Yes

Is the study design appropriate and is the work technically sound?

Yes

Are the conclusions drawn adequately supported by the results?

Partly

Are sufficient details of methods and analysis provided to allow replication by others?

Yes

Reviewer Expertise:

Phytotherapeuics, Clinical Biochemistry, Medical Genetics

I confirm that I have read this submission and believe that I have an appropriate level of expertise to confirm that it is of an acceptable scientific standard.

# Reviewer report: Reviewer response for version 1

**DOI:** 10.5256/f1000research.148054.r228783

**Published:** 2024-05-29

**Authors:** Luis Marquez-Dominguez

**Affiliations:** 1 Instituto Mexicano del Seguro Social, Puebla, Mexico

The quest for compounds exhibiting antiviral activity against diseases such as Zika is of paramount importance, given the absence of a definitive treatment for this ailment. However, I have several suggestions and reservations regarding the manuscript's content. **Among the significant revisions required, it is observed that one of the receptors does not correspond to the Zika virus. Until the authors clarify this discrepancy, the integrity of the results, discussion, conclusions remains questionable.**

 1. In Figure 1, the depiction of structural and non-structural proteins aligns with the complete genome. Therefore, I recommend that the box should encompass the entire genome in the virus diagram, rather than just a segment of it.

 2. Concerning the selection of receptors, the NS5 RdRp with PDB key 5UO4 corresponds to the Structure of human neuronal nitric oxide synthase heme domain in complex with 3-[(2-amino-4-methylquinolin-7-yl)methoxy]-5-(2-(methylamino)ethyl) benzonitrile, and not to the NS5 RdRp of the Zika virus. This discrepancy is also evident in the supplementary data. Was all docking and figure rendering performed using the 5UO4 structure, which does not represent the Zika NS5 RdRp? If so, a substantial portion of the results may not be reflective. I recommend a thorough verification. When compared with NS5 RdRp structures with PDB codes 5Wz3 or 5U0B, there appears to be no similarity.

 3. The methodology mentions obtaining 9 coupling results, yet it fails to specify the criteria used to select the optimal molecular coupling model. Please provide clarity on the selection process.

 4. In the section on "Ligand and receptor molecular docking," the size of the grid is redundantly stated.

 5. In the "Result Molecular Docking" section, the references for the positive ligands (controls) do not support their selection. For example, concerning sinefungin, it is mentioned that they were designed by HVC, but there is no information related to flavivirus or Zika. The same applies to sofosbuvir. I suggest modifying the references to accurately reflect their relevance.

 6. The images exhibit low resolution, hindering effective structure visualization. Therefore, I recommend enhancing clarity for better interpretation. Specifically, in Figure 4, the sharpness is insufficient for clear visualization of 2D diagrams, and the residues interacting with molecules are not distinguishable from the representation of alpha helices and beta sheets. The legend of interactions is unreadable, and the figure caption lacks information on the meaning of each color. For improved comparison, I suggest including controls (sofosbuvir and sinefungin) in Figure 4. Additionally, consider incorporating a surface representation with electrostatic charges to highlight hydrophobic regions or regions with electrostatic charges interacting with molecules.

 7. In Figure 3, in the 5UO4 table, the control -7.40 should be white as it serves as the reference. It appears blue, possibly due to incorrectly taking the positive ligand of 5WXB as a reference. This is inaccurate, as it is a receptor and a distinct molecule.

 8. Table 1 in the docking results mentions different types of non-covalent interactions without justifying why only hydrogen bond bonds and hydrophobic interactions were considered. The discussion also lacks insights into the differences or importance of various interactions such as alkyl-alkyl, pi-alkyl, etc. I recommend ordering the amino acids in ascending order for clarity.

 9. In the discussion of interactions between molecules (rutin) versus sofosbuvir, there is mention of a greater number of interactions, but the explanation on how these interactions lead to blocking the active site is lacking. Consider discussing whether this blocking results from size or other characteristics inherent to enzymatic activity inhibition. A more in-depth discussion of the results is suggested.

Is the work clearly and accurately presented and does it cite the current literature?

Partly

If applicable, is the statistical analysis and its interpretation appropriate?

Not applicable

Are all the source data underlying the results available to ensure full reproducibility?

Partly

Is the study design appropriate and is the work technically sound?

Partly

Are the conclusions drawn adequately supported by the results?

Partly

Are sufficient details of methods and analysis provided to allow replication by others?

Partly

Reviewer Expertise:

virology, antiviral design, rational drug design, and enzyme kinetics, molecular biology.

I confirm that I have read this submission and believe that I have an appropriate level of expertise to state that I do not consider it to be of an acceptable scientific standard, for reasons outlined above.

## References

[1] : Identification of potential inhibitors of Zika virus NS5 RNA-dependent RNA polymerase through virtual screening and molecular dynamic simulations. *Saudi Pharm J* .2020;28(12) : 10.1016/j.jsps.2020.10.005 1580-1591 10.1016/j.jsps.2020.10.005 33424251 PMC7783101

[2] : The dengue virus NS5 protein as a target for drug discovery. *Antiviral Res* .2015;119: 10.1016/j.antiviral.2015.04.010 57-67 10.1016/j.antiviral.2015.04.010 25912817

[3] : Identification and Characterization of Zika Virus NS5 Methyltransferase Inhibitors. *Front Cell Infect Microbiol* .2021;11: 10.3389/fcimb.2021.665379 665379 10.3389/fcimb.2021.665379 33898335 PMC8058401

[4] : Zika virus NS5 protein potential inhibitors: an enhanced in silico approach in drug discovery. *J Biomol Struct Dyn* .2018;36(5) : 10.1080/07391102.2017.1313175 1118-1133 10.1080/07391102.2017.1313175 28351337

[5] : Insights on Dengue and Zika NS5 RNA-dependent RNA polymerase (RdRp) inhibitors. *Eur J Med Chem* .2021;224: 10.1016/j.ejmech.2021.113698 113698 10.1016/j.ejmech.2021.113698 34274831

# Response

**Published:** 2024-06-18

**Authors:** kishore krishna kumaree

**Affiliations:** College of public health, Chulalongkorn University, Bangkok, Bangkok, Thailand

1. In Figure 1, the depiction of structural and non-structural proteins aligns with the complete genome. Therefore, I recommend that the box should encompass the entire genome in the virus diagram, rather than just a segment of it.

**Answer:** Thank you for your suggestion, We have replaced the figure 1 with a new figure in the manuscript.

 2. Concerning the selection of receptors, the NS5 RdRp with PDB key 5UO4 corresponds to the Structure of human neuronal nitric oxide synthase heme domain in complex with 3-[(2-amino-4-methylquinolin-7-yl)methoxy]-5-(2-(methylamino)ethyl) benzonitrile, and not to the NS5 RdRp of the Zika virus. This discrepancy is also evident in the supplementary data. Was all docking and figure rendering performed using the 5UO4 structure, which does not represent the Zika NS5 RdRp? If so, a substantial portion of the results may not be reflective. I recommend a thorough verification. When compared with NS5 RdRp structures with PDB codes 5Wz3 or 5U0B, there appears to be no similarity.

**Answer:**

 Thank you for your meticulous review and valuable feedback. I sincerely apologize for the oversight regarding the receptor used in our study. There was indeed a typographical error in the manuscript. The correct PDB code for the Zika virus NS5 RdRp that we used for our analyses is 5U04, not 5UO4. I assure you that all docking and technical analyses were conducted using the 5U04 structure, which accurately represents the Zika NS5 RdRp. The incorrect PDB code in the text was an unfortunate typographical mistake, and we will promptly correct this in the manuscript and the supplementary data to reflect the accurate PDB code.

 We greatly appreciate your diligence in pointing out this discrepancy and apologize for any confusion it may have caused. Your feedback is crucial for maintaining the integrity of our work, and we will ensure that such errors are rectified to convey our research findings accurately.

 3. The methodology mentions obtaining 9 coupling results, yet it fails to specify the criteria used to select the optimal molecular coupling model. Please provide clarity on the selection process.

**Answer:**

 The selection of the optimal molecular model was done based on the lower binding energy (more negative value), a higher number of hydrogen bonds, and other nonbonded interactions, including hydrophobic and electrostatic interactions. In addition, conserved binding pocket interactions were also considered and compared with the known control inhibitors for the respective target proteins.We believe this multifaceted approach provides a more robust optimal model selection.

 4. In the section on "Ligand and receptor molecular docking," the size of the grid is redundantly stated.

**Answer:**The redundancy mentioning of grid size has been corrected in the revised manuscript.

 5. In the "Result Molecular Docking" section, the references for the positive ligands (controls) do not support their selection. For example, concerning sinefungin, it is mentioned that they were designed by HVC, but there is no information related to flavivirus or Zika. The same applies to sofosbuvir. I suggest modifying the references to accurately reflect their relevance.

**Answer:**

 Thank you for your careful review and for highlighting the need to ensure the references for our positive control ligands are accurate and relevant. We apologize for any oversight in the current references and appreciate your suggestion to provide more relevant citations.

 In response to your concern and to support our selection, we have updated the manuscript and have included the following references:

 Sinefungin and Sofosbuvir were the reference inhibitors for NS5-MTase and NS5 RdRp, respectively.

References:

- Hercik, K., et al., *Structural basis of Zika virus methyltransferase inhibition by sinefungin.* Archives of virology, 2017. **162**: p. 2091-2096.
- Lin, Y., et al., *Identification and characterization of Zika virus NS5 RNA-dependent RNA polymerase inhibitors.* International journal of antimicrobial agents, 2019. **54**(4): p. 502-506.
-  Sacramento, C.Q., et al., *The clinically approved antiviral drug sofosbuvir inhibits Zika virus replication.* Scientific reports, 2017. **7**(1): p. 40920.
- Tao, Z., et al., *Design, synthesis and in vitro anti-Zika virus evaluation of novel Sinefungin derivatives.* European Journal of Medicinal Chemistry, 2018. **157**: p. 994-1004.

6. The images exhibit low resolution, hindering effective structure visualization. Therefore, I recommend enhancing clarity for better interpretation. Specifically, in Figure 4, the sharpness is insufficient for clear visualization of 2D diagrams, and the residues interacting with molecules are not distinguishable from the representation of alpha helices and beta sheets. The legend of interactions is unreadable, and the figure caption lacks information on the meaning of each color. For improved comparison, I suggest including controls (sofosbuvir and sinefungin) in Figure 4. Additionally, consider incorporating a surface representation with electrostatic charges to highlight hydrophobic regions or regions with electrostatic charges interacting with molecules.

**Answer:**

 Thank you for your constructive feedback regarding the visualization quality of the images in Figure 4. We have considered your recommendations and replaced the images with higher-resolution versions to enhance clarity and facilitate better interpretation. However, at this stage, we have not incorporated surface representations with electrostatic charges to highlight hydrophobic regions and regions with electrostatic interactions. We will consider including these enhancements in future revisions to improve the visualization quality further.

 Additionally, we have included controls (sofosbuvir and sinefungin) in Figure 4 for improved comparison. We appreciate your valuable feedback, which has significantly improved the quality and clarity of our manuscript. Thank you for your continued support in refining our research.

 7. In Figure 3, in the 5UO4 table, the control -7.40 should be white as it serves as the reference. It appears blue, possibly due to incorrectly taking the positive ligand of 5WXB as a reference. This is inaccurate, as it is a receptor and a distinct molecule.

**Answer:**

 Thank you for pointing out the issue with the color assignment in Figure 3. We have redone the color mapping for the complexes 5U04 and 5WXB table in Figure 3. The new figure has been added to the manuscript.

 8. Table 1 in the docking results mentions different types of non-covalent interactions without justifying why only hydrogen bond bonds and hydrophobic interactions were considered. The discussion also lacks insights into the differences or importance of various interactions such as alkyl-alkyl, pi-alkyl, etc. I recommend ordering the amino acids in ascending order for clarity.

**Answer:**

 Thank you for your insightful feedback regarding the non-covalent interactions mentioned in Table 1 of the docking results.

  In our study, we focused on hydrogen bonds and hydrophobic interactions due to their significant roles in stabilizing ligand binding within the receptor's active site. Hydrogen bonds are essential for providing specificity and strong directional interactions, while hydrophobic interactions contribute to binding affinity by facilitating the burial of non-polar surfaces and reducing desolvation penalties. Although other non-covalent interactions such as alkyl-alkyl, pi-alkyl, and pi-pi interactions also play important roles in ligand-receptor binding, we prioritized hydrogen bonds and hydrophobic interactions due to their dominant influence in many docking studies. We have also included an explanation in the manuscript's discussion section.

 Furthermore, we have arranged the amino acids in Table 1 in their chronological order of occurrence within the protein sequence and structure, reflecting the sequential interaction pattern and spatial context, which is crucial for understanding the binding interactions. This arrangement aids in visualizing the interactions as they occur along the protein's active site and aligns with standard conventions in protein interaction studies.

 9. In the discussion of interactions between molecules (rutin) versus sofosbuvir, there is mention of a greater number of interactions, but the explanation on how these interactions lead to blocking the active site is lacking. Consider discussing whether this blocking results from size or other characteristics inherent to enzymatic activity inhibition. A more in-depth discussion of the results is suggested.

**Answer:**

 Thank you for your valuable feedback and suggestion for a more in-depth discussion of the interactions between rutin and sofosbuvir

 To address the interactions between rutin and sofosbuvir with the active site, we provide a more detailed discussion on how these interactions contribute to the inhibition of enzymatic activity. Rutin exhibits a greater number of interactions compared to sofosbuvir, which enhances its ability to effectively block the active site. This blocking is not solely a function of the number of interactions but also their nature and strength. The larger size of rutin allows it to span a broader area of the active site, facilitating multiple points of contact through hydrogen bonds, hydrophobic interactions, and aromatic stacking. These interactions collectively stabilize the binding of rutin within the active site, thereby preventing substrate access and subsequent enzymatic activity. In contrast, while sofosbuvir also forms stable interactions, the fewer number and potentially weaker nature of these interactions may result in less effective blocking. Thus, rutin's increased binding affinity and comprehensive interaction profile contribute to its potential as a more effective inhibitor of the enzyme's active site.

# Reviewer report: Reviewer response for version 1

**DOI:** 10.5256/f1000research.148054.r214337

**Published:** 2023-10-18

**Authors:** Arumugam Vijaya Anand

**Affiliations:** 1 Department of Human Genetics and Molecular Biology, Bharathiar University, Coimbatore, India

The authors have put effort to evaluate the “ *In silico* screening for potential inhibitors from the phytocompounds of *Carica papaya* against Zika virus NS5”. The authors describe the research undertaken with this in an organized manner, emphasizing the results obtained by them. The article needs modification for better cohesion of information to achieve the goal and the shortcomings which need to be considered. Hence, the paper can be approved after MAJOR REVISIONS are carried out.

 1. The English need improvement since there are some grammatical and syntax errors in the manuscript. For example, the words “subjected for” may be as “subjected to”; “NS5-MTase” as “the NS5-MTase”; “by ADMET” as “by an ADMET”; “addition of” as “the addition of”; “removal of” as “the removal of”; “ranging 0.111” as “ranging from 0.111”; “zeaxanthin” as “and zeaxanthin”. The grammar mistakes which are not mentioned here are also to be checked and corrected properly.

 2. There are some typing mistakes as well, and authors are advised to carefully proof-read the text. For example, the words “encode a” may be as “encodes a”; “decent” as “descent”; “Ligands” as “Ligand”; “best docked” as “best-docked”; “nor vaccines” as “no vaccines”; “other site” as “other side”; “rutin, has” as “rutin has”; “β-sitsterol” as “β-sitosterol or check correct spelling”; “caretenoids” as “carotenoids”. The typos not mentioned here are also to be checked and corrected properly.

 3. Check the abbreviations throughout the manuscript and introduce the abbreviation when the full word appears the first time in the abstract (The use of abbreviations in the abstract (ADME) section may distract readers who wish to quickly skim through several publications before deciding to read one in full) and the remaining part of the manuscript (For example, PDP, FDA, etc.,). Make a word abbreviated in the article that is repeated at least three times in the text, not all words to be abbreviated.

 4. The full form of the species should be given when the first time appears in both the abstract and in the remaining part of the manuscript and it should be followed by only the first letter of the genus (For example, *Carica papaya *when the first time appear and followed by *C. papaya*).

 5. The authors should use uniformly either “ZIKA” or “Zika” all over the manuscript for better understanding.

 6. The authors may cite recent prevalence or incidence data Zika virus (ZIKV) infection and it should be at-least of 2022 or 2023.

 7. The introduction part appears less informative about *Carica papaya*, thus this section should be indicated as detailed to understand the manuscript in clear since the main objectives is focused on *Carica papaya*. The botanical description, family name and different plants of the plant with it medicinal uses.

 8. The authors should rewrite the following “The paper aims” as “The present work or study aims”.

 9. The authors should avoid the repetition some content in the manuscript and also it affect the flow of reading. It should be carefully checked and removed. For example, the family name “Flaviviridae” of ZIKA has been mentioned in three times.

 10. The conclusion seems in general. All conclusions must be convincing statements on what was found to be novel, impact based on the strong support of the review. Moreover, the authors may also be included the limitation of the present findings for a better understanding of the manuscript.

Is the work clearly and accurately presented and does it cite the current literature?

Yes

If applicable, is the statistical analysis and its interpretation appropriate?

Not applicable

Are all the source data underlying the results available to ensure full reproducibility?

Yes

Is the study design appropriate and is the work technically sound?

Yes

Are the conclusions drawn adequately supported by the results?

Partly

Are sufficient details of methods and analysis provided to allow replication by others?

Yes

Reviewer Expertise:

Phytotherapeuics, Clinical Biochemistry, Medical Genetics

I confirm that I have read this submission and believe that I have an appropriate level of expertise to confirm that it is of an acceptable scientific standard, however I have significant reservations, as outlined above.

# Response

**Published:** 2024-06-18

**Authors:** kishore krishna kumaree

**Affiliations:** College of public health, Chulalongkorn University, Bangkok, Bangkok, Thailand

1. The English need improvement since there are some grammatical and syntax errors in the manuscript. For example, the words "subjected for" may be as "subjected to"; "NS5-MTase" as "the NS5-MTase"; "by ADMET" as "by an ADMET"; "addition of" as "the addition of"; "removal of" as "the removal of"; "ranging 0.111" as "ranging from 0.111"; "zeaxanthin" as "and zeaxanthin". The grammar mistakes which are not mentioned here are also to be checked and corrected properly.

**Answer: **Thank you for your detailed feedback regarding the grammatical and syntax errors in the manuscript. We have thoroughly reviewed the entire manuscript to identify and correct any other grammatical and syntax errors that were not mentioned.

 2. There are some typing mistakes as well, and authors are advised to carefully proof-read the text. For example, the words "encode a" may be as "encodes a"; "decent" as "descent"; "Ligands" as "Ligand"; "best docked" as "best-docked"; "nor vaccines" as "no vaccines"; "other site" as "other side"; "rutin, has" as "rutin has"; "β-sitsterol" as "β-sitosterol or check correct spelling"; "caretenoids" as "carotenoids". The typos not mentioned here are also to be checked and corrected properly.

**Answer:** Thank you for your detailed feedback 4in the manuscript. We have thoroughly reviewed the entire manuscript to manage necessary corrections.

 3. Check the abbreviations throughout the manuscript and introduce the abbreviation when the full word appears the first time in the abstract (The use of abbreviations in the abstract (ADME) section may distract readers who wish to quickly skim through several publications before deciding to read one in full) and the remaining part of the manuscript (For example, PDP, FDA, etc.,). Make a word abbreviated in the article that is repeated at least three times in the text, not all words to be abbreviated.

**Answer: **Thank you for your suggestion. We have reviewed the manuscript to ensure that abbreviations are introduced upon first use and are only applied to terms repeated at least three times in the text, to maintain clarity and readability.

 4. The full form of the species should be given when the first time appears in both the abstract and in the remaining part of the manuscript and it should be followed by only the first letter of the genus (For example, Carica papaya when the first time appear and followed by C. papaya).

**Answer:** Thank you for your suggestion. The corrections are made in the manuscript.

 5. The authors should use uniformly either "ZIKA" or "Zika" all over the manuscript for better understanding.

**Answer:** Thank you for your feedback. We have revised the manuscript to use "Zika" uniformly throughout the text for consistency and better understanding.

 6. The authors may cite recent prevalence or incidence data Zika virus (ZIKV) infection and it should be at-least of 2022 or 2023.

**Answer**: Thank you for your suggestion. We have updated the manuscript to include recent prevalence and incidence data on Zika virus infections from 2022 and 2023 to provide current and relevant information.

 7. The introduction part appears less informative about Carica papaya, thus this section should be indicated as detailed to understand the manuscript in clear since the main objectives is focused on Carica papaya. The botanical description, family name and different plants of the plant with it medicinal uses.

**Answer:** Thank you for your kind suggestion. We have updated the manuscript on the medicinal and botanical significance of papaya.

 8. The authors should rewrite the following "The paper aims" as "The present work or study aims".

**Answer:** Thank you for your suggestions. We have updated the manuscript.

 9. The authors should avoid the repetition some content in the manuscript and also it affect the flow of reading. It should be carefully checked and removed. For example, the family name "Flaviviridae" of ZIKA has been mentioned in three times.

**Answer: **Thank you for your feedback. We have carefully reviewed the manuscript and have reduced the redundant mentions of the family name "Flaviviridae" to improve clarity and coherence.

 10. The conclusion seems in general. All conclusions must be convincing statements on what was found to be novel, impact based on the strong support of the review. Moreover, the authors may also be included the limitation of the present findings for a better understanding of the manuscript.

**Answer:** Thank you for your valuable feedback. We have revised the conclusion to highlight the novel findings and their impact, ensuring that our statements are well-supported by the review. Additionally, we will include the limitations of the present study, such as the reliance on in silico methods, which need further validation through experimental and clinical studies.
